# Change in Quality of Life in Patients with Advanced Rectal Cancer Between 2010 and 2022

**DOI:** 10.3390/healthcare12212108

**Published:** 2024-10-23

**Authors:** Ailina Doelz, Daniel Blasko, Claudia Schweizer, Tim Fitz, Annett Kallies, Rainer Fietkau, Luitpold Distel

**Affiliations:** 1 Department of Radiation Oncology, Universitätsklinikum Erlangen, Friedrich-Alexander-Universität Erlangen-Nürnberg (FAU), 91054 Erlangen, Germany; ailina.doelz@fau.de (A.D.); dblasko922@gmail.com (D.B.); claudia.schweizer@uk-erlangen.de (C.S.); tim.fitz@uk-erlangen.de (T.F.); annett.kallies@uk-erlangen.de (A.K.); rainer.fietkau@uk-erlangen.de (R.F.); 2 Comprehensive Cancer Center Erlangen-EMN (CCC ER-EMN), Universitätsklinikum Erlangen, Friedrich-Alexander-Universität Erlangen-Nürnberg (FAU), 91054 Erlangen, Germany

**Keywords:** health-related quality of life, QLQ-C30, rectal cancer, long-term follow-up, radiochemotherapy, neoadjuvant

## Abstract

Background/Objectives: Advanced rectal cancer is one of the most common cancers worldwide and has a significant impact on public health. Because favorable and long-term survival has been achieved with multimodal therapy, patient quality of life is very important. The intention of this study was to assess patients’ quality of life using various functioning and symptom scores from the years 2010 to 2022 and to examine changes over time. Methods: Data on health-related quality of life were collected from rectal cancer patients treated at the University Hospital Erlangen in Germany over a period of 13 years. The EORTC QLQ-C30 questionnaire and the rectal cancer-specific module QLQ-CR38 were completed in this study by a total of 516 patients. The questionnaires were collected before, during and at annual follow-up visits after treatment. Statistical significance was defined as *p*-values < 0.05 as well as a difference of 10 or more percentage points. Results: The deterioration in scores is most pronounced immediately after radiochemotherapy, especially for pain (+19.8 pp), fatigue (+16.1 pp) and diarrhoea (+24.8 pp). One year after the end of therapy, most of the values are again comparable to or better than those of the German general population and only role functioning (−19.8 pp), social functioning (−24.6 pp), diarrhoea (−21.6 pp) and financial difficulties (−16.3 pp) are considerably worse. Some baselines deteriorate clearly over time from 2010 to 2022; these are role functioning (−23.9 pp), social functioning (−17.3 pp), body image (−15.2 pp), fatigue (+13.8 pp) and nausea and vomiting (+10.5 pp). Conclusions: An improvement in therapy in terms of a reduction in side effects and, thus, an improvement in quality of life over time could not be proven. The deterioration in individual scores over time does not appear to be a problem specific to rectal cancer patients, but rather, is associated with social developments or systemic healthcare factors in German society that are not directly related to oncological diseases.

## 1. Introduction

Rectal cancer (RC) is one of the most frequent cancers in the world and it accounts for approximately one-third of all colorectal cancer (CRC) cases. With over 1.9 million new cases and 904,000 deaths estimated in 2022, CRC represents nearly one-tenth of all cancer cases and deaths. Despite ranking third in incidence, CRC ranks second in mortality [1], emphasizing the need for health initiatives directed towards enhancing access to high-quality healthcare [2]. Surgery, chemotherapy and radiotherapy are the three most commonly used methods in clinical practice for the treatment of RC [3] and have significantly improved long-term survival, with 10-year overall survival rates ranging from 55.7% to 64.7% [4]. Here, the most important developments have been the introduction of total mesorectal excision (TME), minimally invasive surgery, neoadjuvant (pre-operative) therapy, magnetic resonance imaging (MRI) and standardized histopathological assessment [5]. Thanks to a wide range of customization options in the treatment of rectal cancer, there is a highly individualized therapy for each specific case [6]. However, these treatments can detrimentally affect patients’ quality of life (QOL) during therapy. Understanding and improving QOL for RC patients is crucial for holistic cancer care. The concept of health-related quality of life can be defined as a measurement of the physical, psychological and social effects of illness or treatment [7,8]. To assess QOL in cancer patients, the European Organization for Research and Treatment of Cancer (EORTC) has developed the QLQ-C30 questionnaire [9] and the module tailored for colorectal cancer QLQ-CR38 [10], providing reliable evaluations of patients’ QOL. In this study, these two questionnaires were given to the patients as a single unit and were always completed simultaneously and in full. Given the general association between higher QOL and lower mortality rates [11], our results can additionally provide indications of the efficacy of the treatment and its impact on side effects.

Technological improvements in radiation therapy have improved in precision and accuracy during the last decade [12,13], prompting the question regarding to what extent the QOL of RC patients is affected by these changes. The aim of this study was to examine the changes over time and how they correlate with the patients’ QOL during and after treatment. The novelty of our study is that the quality of life of patients with a specific disease, namely advanced rectal cancer, has been continuously monitored over a very long period of 13 years with the same neoadjuvant treatment strategy, but with continuous improvements in the different components of the therapy.

## 2. Materials and Methods

This study included 516 patients with advanced rectal cancer. They were treated with radiochemotherapy at the University Hospital Erlangen, Germany. In total, 75% of the surgeries took place in the university hospital and 25% in peripheral hospitals. Eligibility for the trial was based on planned radiotherapy for rectal cancer. All patients who were offered the therapy were asked whether they would like to take part in the study. The patients received neoadjuvant radiochemotherapy followed by surgical excision of the mesorectum, corresponding to a total mesorectal excision (TME) [14,15,16,17]. A total of 139 patients received a stoma during their treatment. Most patients in our clinic received low anterior resection of the rectum with a descendo-rectostomy performed with a stapled end-to-end anastomosis. All participants of the study provided written consent prior to their involvement, allowing the collection of their clinical data. The collection of data took place from May 2010 to September 2022. The questionnaires by the European Organization for Research and Treatment of Cancer (EORTC) used for this study, QLQ-C30 and QLQ-CR38, included a total of 68 questions, with response options on a scale from 1 to 4, with “1 = not at all” to “4 = very much”, with two exceptions ranging from “1 = very poor” to “7 = excellent”. Nineteen questions referred to different subgroups (male/female or patients with/without a stoma), and the remaining forty-nine questions referred to all included patients. The answers to the questionnaires were summarized into 27 scores which each included one to seven questions. Only 23 out of 27 scores were used in this study due to a lack of data for subgroups and/or questions which included private sexual questions. Therefore, 14 symptom scores and nine functional scores were used in the analysis. All mean scores of the EORTC questionnaire subscales were recalculated and are shown as percentages ranging from 0 to 100%. This resulted in functional scores with a higher percentage and symptom scores with a lower percentage being more desirable, thus indicating better QOL.

**Figure 1:**
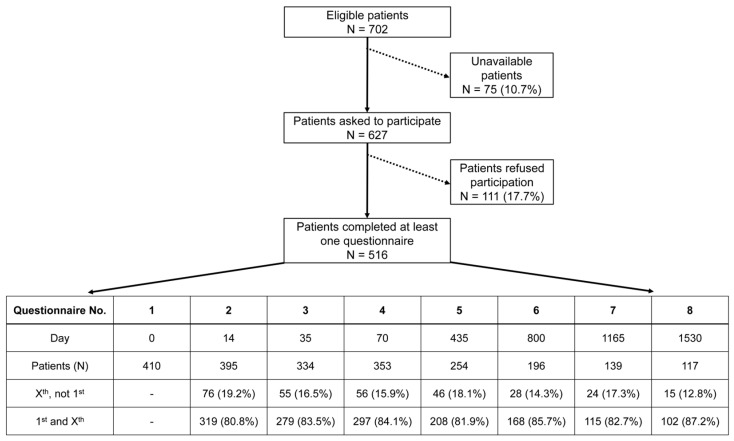
A flowchart depicting the progress of patient recruitment leading to 516 study participants. Unavailable patients were those who, despite several attempts to obtain consent for this study, could not be contacted before the start of treatment. The table lists the descriptive statistics of questionnaire Nos. 1–8. The number of patients for each questionnaire is further broken down into two categories: X^st^, not 1st = those who completed a subsequent questionnaire without answering the initial one; 1st and X^st^ = those who completed the initial and a subsequent questionnaire.

The questionnaires were handed out at eight different time points accompanying each patient’s therapy: at the beginning of (questionnaire 1; day 0), during (questionnaire 2; day 14) and at the end of (questionnaire 3; day 35) radiochemotherapy, shortly before surgery (questionnaire 4; day 70), and at yearly follow-up time points thereafter for four years: one year (questionnaire 5; day 435), two years (questionnaire 6; day 800), three years (questionnaire 7; day 1165), four years (questionnaire 8; day 1530). Every patient received their questionnaire either in person during radiotherapy or through mail after finishing their treatment. In the process of patient recruitment, every patient receiving treatment for advanced rectal cancer in our facility was invited to participate in this study. A total of 516 patients answered at least one questionnaire (Figure 1). Not all patients decided to complete every questionnaire, which resulted in some answering the first and missing one or more consecutive questionnaires or others missing the first. The criterion for canceling a patient’s survey was if they refused to be interviewed further or did not respond to two consecutive surveys by mail. The reason for non-participation or cancelation by the patient was not asked. This, along with different and decreasing response rates, as well as the deaths of patients, resulted in varying sample sizes for each questionnaire.

Within the scope of this study, the emphasis was placed on the first five questionnaires. Baseline questionnaire 1, end of RCT questionnaire 3 and 1-year follow-up questionnaire 5 were selected to examine changes in individual scores over time. We have included linear regression lines to provide an interpretable way of understanding the relationships between variables and to minimize discrepancies in values. The age of the participants at the beginning of treatment (day 0) ranged from 15 to 93 years, with a mean age of 63.6 years. Among the 516 patients surveyed, 357 were male, while 159 were female. Each patient was allocated to a group according to the year when they answered their first questionnaire, resulting in a total of 13 individual groups from 2010 to 2022. Over the 13 years, an average of 40 patients per year were included, with a range of 20 to 55 patients per year.

The participants were interviewed successively, and quality-of-life data were collected in a prospective manner, while clinical information was obtained retrospectively from patient records. The responses were entered into an Excel 2016 spreadsheet and analyzed using custom code written in Visual Basic for Applications (VBA). The categorization into groups from 2010 to 2022 was carried out in Excel 2024. GraphPad Prism 10.02.0 was used for data analyses of *p*-values, linear regressions, means, medians and percentiles. Statistical significance was defined as a *p*-value < 0.05 as well as a difference of 10 or more percentage points (pp), according to the suggestion of Osoba et al., who defined differences of 10 percentage points or more as clinically relevant [18].

## 3. Results

### 3.1. Cohort of 516 Rectal Cancer Patients

The sample consisted of a total of 516 patients who were surveyed before, during and after receiving radiochemotherapy for advanced rectal cancer. The characteristics of all participants concerning age, gender, TNM-staging, UICC-staging and grading are shown in the following table (Table 1). The participants were assigned into groups from 2010 to 2022, according to the year the first EORTC questionnaire was answered. The response and participation rates were highest in 2013 and lowest in 2010, as data collection had only just started in May of 2010. Concerning age, the youngest patient was 15 years old in 2019, while the oldest patient was 93 years old in 2022. The mean age varied from 60.2 to 66.5 years over the years. Male patients were generally more highly represented (mean 69.2%), except for the year 2022, where women had a share of 52%, and men 48%.

### 3.2. Symptom and Functional Score Distribution of Quality of Life

**Figure 2:**
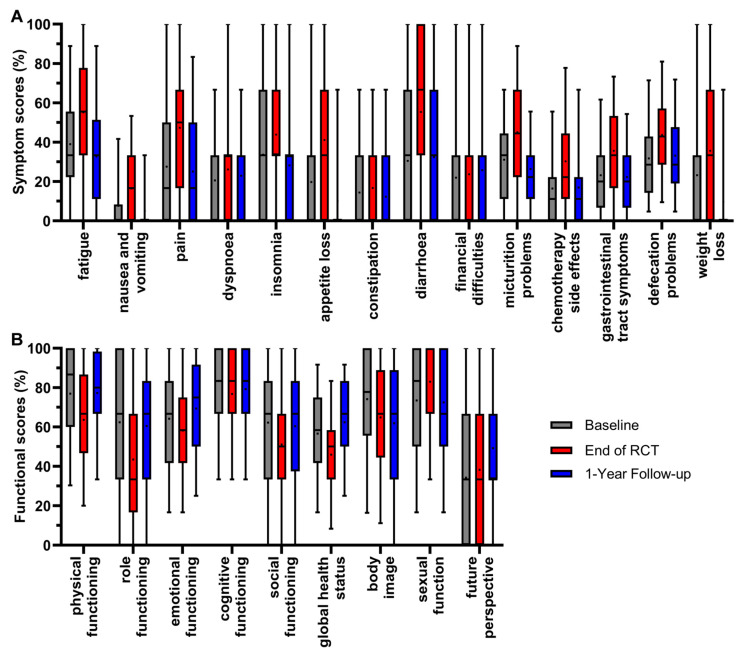
Boxplots of symptom (**A**) and functional scores (**B**) of all patients from 2010 to 2022, and comparison of baseline, end of RCT and 1-year follow-up, with whiskers defined as 5th and 95th percentiles. Means are shown as dots. If boxplots are not displayed, it indicates that more than 75% of the data points have a value of 0%.

Twenty-three scores of all patients participating in the survey were analyzed between 2010 and 2022 at three representative time points of the RC patients’ course of treatment. These were immediately before the start of radiochemotherapy (RCT) (day 0/baseline), at the end of radiochemotherapy (day 35) and at the first annual follow-up (day 435) (Figure 2).

The scores were determined by the fact that the patients underwent radiochemotherapy followed by major surgery. The mean value of all symptom scores (Figure 2A) was highest at the end of radiochemotherapy, except for financial difficulties. The mean value of appetite loss first rose at the end of RCT (+21.4 pp), and then decreased at the 1-year follow-up (−31.1 pp); the same occurred with pain (+19.8 pp/−22.2 pp), fatigue (+16.1 pp/−22.4 pp), micturition problems (+14.0 pp/−18.8 pp), weight loss (+12.4 pp/−25.8 pp), gastrointestinal tract symptoms (+12.4 pp/−13.4 pp), insomnia (+10.2 pp/−15.6 pp) and nausea and vomiting (+8.9 pp/−11.4 pp). The score for constipation followed this pattern, rising by 2.3 pp and then decreasing by 4.5 pp, while the changes were minor compared to the previous scores. Significant changes in symptom scores resulting in the most favorable outcome of a lower score compared to baseline at the 1-year follow-up therefore occurred for the symptom scores of fatigue, nausea and vomiting, pain, insomnia, appetite loss, micturition problems, weight loss and gastrointestinal tract symptoms. For the symptom scores of dyspnoea, diarrhoea, chemotherapy side effects and defecations problems, the mean value of the first annual follow-up ranged between baseline and the end of RCT, meaning that symptoms decreased while remaining only slightly higher than baseline: the symptom score for diarrhoea increased by 24.8 pp and then decreased by 22.7 pp at the 1-year follow-up. Similarly, the scores for chemotherapy side effects (+13.8 pp/−13.3 pp), defecation problems (+11.9 pp/−10.5 pp) and dyspnoea (+5.6 pp/−3.3 pp) increased at the end of RCT and decreased at the 1-year follow-up. The mean value of financial difficulties was the only symptom score, which slightly, not significantly, ameliorated throughout all three time points by 1.8 pp after RCT and 2.1 pp at the 1-year follow-up.

For the functional scores (Figure 2B), a higher score is more favorable and best results for patients are rising scores over time. Again, the mean values were lowest after RCT for most scores, with significance for physical functioning, dropping (−13.3 pp), and thereafter increasing (+13.7 pp) to an equal mean value at the first year of follow-up compared to baseline. The same trend could be seen for global health status (−10.7 pp/+16.5 pp), emotional functioning (−6.0 pp/+11.2 pp), role functioning (−18.9 pp/+17.0 pp) and social functioning (−11.1 pp/+9.3 pp). The changes during treatment and follow-up for cognitive functioning (−6.7 pp/+2.5 pp) were minor and not significant, indicating generally small effects on cognitive functioning in RC treatment. Contrary to the overall trend, sexual function tended to rise (+9.6 pp) at the end of RCT and clearly decreased (−10.6 pp) thereafter at the 1-year follow-up. The only functional score with a trend of constantly decreasing mean values over the course of the three time points was body image (−9.3 pp/−3.0 pp). In contrast, the score for future perspective showed a minor increase after RCT (+4.1 pp) and a distinct increase at the 1-year follow-up (+15.0 pp) compared to baseline, making it the only functional score with consistent improvement.

### 3.3. Clear Changes in Quality of Life over 13 Years

**Figure 3:**
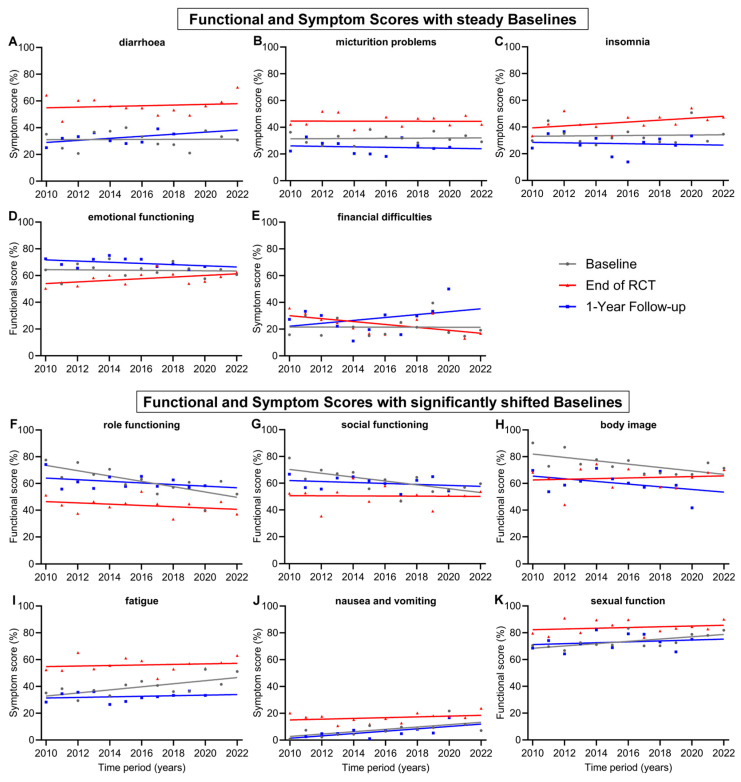
Means and regression lines of functional and symptom scores with steady baselines (**A**–**E**) and significantly shifted baselines (**F**–**K**) from year 2010 to 2022. The subfigures display the scores: (**A**) diarrhoea, (**B**) micturition problems, (**C**) insomnia, (**D**) emotional functioning, (**E**) financial difficulties, (**F**) role functioning, (**G**) social functioning, (**H**) body image, (**I**) fatigue, (**J**) nausea and vomiting, (**K**) sexual function. Baseline (day 0) compared to end of RCT (radiochemotherapy) (day 35) and to 1-Year follow-up (day 435).

In addition to presenting a general evolution of QOL for all patients during treatment for advanced rectal cancer, we were also interested in changes in individual scores over time, as new technologies in radiation therapy, drugs to treat cancer or its side effects and new treatment plans have been developed. Items with relatively steady baselines and only with minor changes over the 13-year time span of this study were diarrhoea (+0.4 pp), micturition problems (+0.7 pp), insomnia (+1.1 pp), emotional functioning (−1.0 pp) and financial difficulties (−0.2 pp) (Figure 3A–E). However, there were functional and symptom scores that experienced significantly shifted baselines from 2010 to 2022. The largest decrease was in role functioning (−23.9 pp), followed by social functioning (−17.3 pp) and body image (−15.2 pp). Significant worsening could be seen for the symptom scores of fatigue (+13.8 pp) and nausea and vomiting (+10.5 pp) (Figure 3F–J). Further, the score for nausea and vomiting increased significantly (+10.5 pp) in the regression line of the 1-year follow-up, similar to the increase in baseline regression. However, changes during every other time point in treatment, including during RCT, after RCT, and before and after surgery, were not significant. These findings indicate that prior to treatment, the participants felt significantly worse in their role functioning, social functioning and body image and experienced more fatigue, nausea and vomiting over the years. The baseline regression line of the score for sexual function (Figure 3K) was the only increasing functional score (+10.3 pp) and, therefore, a significant improvement for patients. During treatment, there were generally few significant positive changes in the participants’ QOL. These included fewer financial difficulties at the end of RCT (−13.1 pp) and preoperative sexual functionality (+11.4 pp).

**Figure 4:**
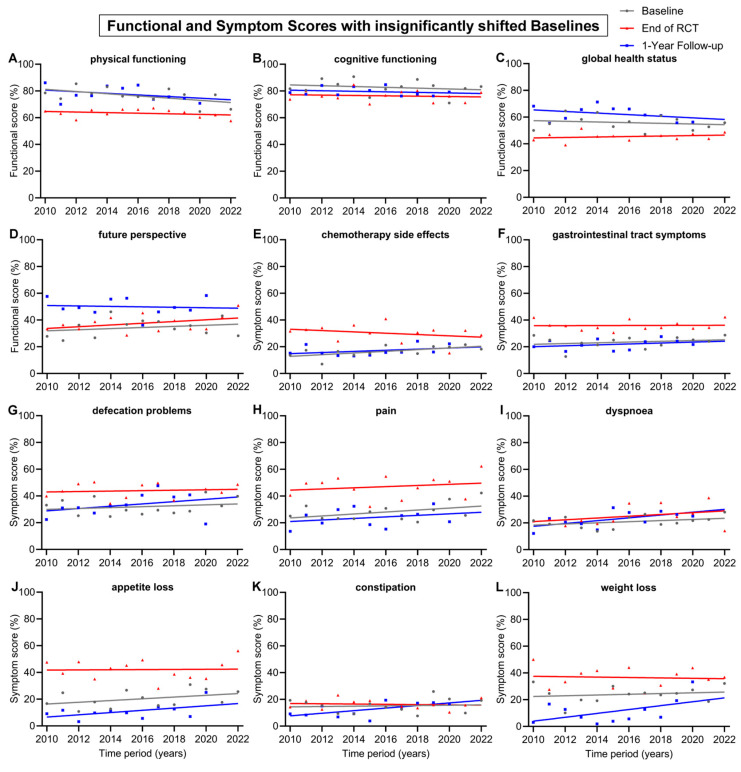
Means and regression lines of functional and symptom scores with insignificant shifts in baseline from 2010 to 2022. The subfigures display the scores: (**A**) physical functioning, (**B**) cognitive functioning, (**C**) global health status, (**D**) future perspective, (**E**) chemotherapy side effects, (**F**) gastrointestinal tract symptoms, (**G**) defecation problems, (**H**) pain, (**I**) dyspnoea, (**J**) appetite loss, (**K**) constipation and (**L**) weight loss. Baseline (day 0) compared to end of RCT (radiochemotherapy) (day 35) and to 1-Year follow-up (day 435).

Noteworthy are the trends of a significant approximation of both regression lines at baseline and the end of RCT for the scores for role functioning, social functioning, body image, fatigue and chemotherapy side effects (Figure 3F–I and Figure 4E). In 2010, the gap between baseline and the end of RCT regression line of the score for role functioning was 27.1 pp, while in 2022, the gap was only 9.0 pp, receding by 18.1 pp. Likewise, the regression lines for body image converged, resulting in a gap reduction of 18.2 pp (19.4 to 1.2 pp) between 2010 and 2022. Similarly, the scores for social functioning converged by 16.8 pp (19.7 to 2.9 pp), for fatigue by 11.3 pp (22.0 to 10.7 pp) (Figure 3) and for chemotherapy side effects (Figure 4E) by 13.5 pp (20.3 to 6.8 pp). Financial difficulties was the only score where the regression lines of baseline and end of RCT crossed over, resulting in a distinct difference of 12.9 pp from 2010 to 2022, marking fewer financial difficulties at the end of RCT than before the start of treatment (Figure 3E). There were additional scores that showed tendencies towards converging regression lines, although these tendencies were not as pronounced as those previously mentioned: these were emotional functioning, converging by 8.3 pp, insomnia by 7.6 pp, physical functioning by 7.3 pp, nausea and vomiting by 7.1 pp, appetite loss by 7.1 pp and sexual function by 7.0 pp.

### 3.4. Quality-of-Life Scores for Which There Was Hardly Any Change over 13 Years

Other baseline and 1-year follow-up regression lines were largely parallel, such as body image (Figure 3H), nausea and vomiting (Figure 3J), physical functioning (Figure 4A), chemotherapy side effects (Figure 4E), gastrointestinal tract symptoms (Figure 4F), pain (Figure 4H) and appetite loss (Figure 4J).

The remaining 12 scores had insignificant shifts (<10 pp) in baseline from 2010 to 2022 (Figure 4). The regression lines of baseline all tended to decrease, except for the score for future perspective, which tended to rise by 5.0 pp. There was also only a small change in the regression lines at the end of RCT. However, over the 13-year time span, the regression line of the 1-year follow-up increased clearly for the symptom scores for weight loss (+17.4 pp), dyspnoea (+12.7 pp), constipation (+11.6 pp), defecation problems (+10.4 pp) and appetite loss (+10.1 pp). The 1-year follow-up regression lines generally increased for symptom scores and decreased for functional scores. Likewise, this was also the case for the regression line of end of RCT, which tended to increase in every symptom score, except constipation (−1.2 pp), chemotherapy side effects (−5.9) and weight loss (−1.8 pp). The only functional scores which tended to rise at the end of RCT were global health status (+2.1) and future perspective (+7.8 pp); every other functional score slightly decreased.

### 3.5. Is There a Deterioration in Clinical Characteristics over Time in the Patient Cohort?

**Figure 5:**
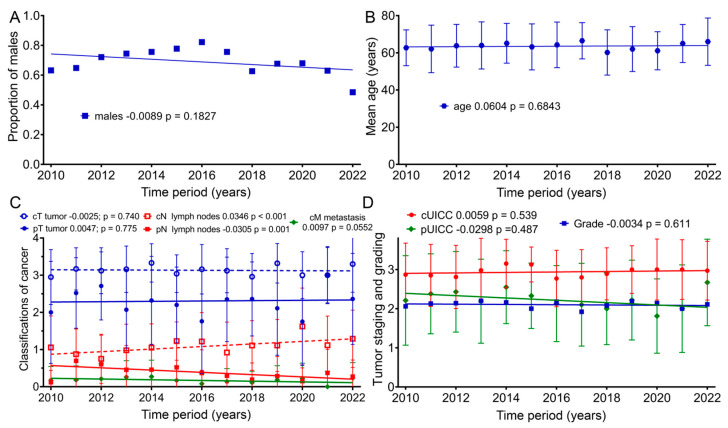
Means and regression lines of patients’ (**A**) proportion of males, (**B**) age, (**C**) TNM stage and (**D**) grading and UICC from 2010 to 2022. The first number after the designation indicates the slope of the regression per year, and the second number the significance of the correlation.

Because there was a decrease in baseline for many of the scores over time, we examined whether there was a change in age, gender, tumor stage or grade. Therefore, further analyses of the clinical characteristics of the patient groups regarding age, as well as TNM (Tumor Node Metastasis) and UICC (Union for International Cancer Control) staging, were conducted (Figure 5): within our sample group, the patients’ mean age at the beginning of therapy did not clearly change from 2010 to 2022 (*p* = 0.183). Nevertheless, for the first time, females were slightly more represented than males (48%) in 2022. The mean age of patients according to the regression line slightly increased by 0.7 years from 2010 (63.2 years) to 2022 (63.9 years) (*p* = 0.684). From 2010 to 2022, the regression lines of the mean values for clinical TNM staging regarding tumor size (cT) remained largely unchanged, with a slope of −0.0025 per year (*p* = 0.740). Lymph node staging (cN) rose significantly, with a slope of 0.0346 per year (*p* < 0.001). Clinical staging for distant metastasis (cM) increased, with an annual slope of 0.0097 (*p* = 0.055). Pathological TNM staging from 2010 to 2022 increased only slightly for tumor size (pT) by an annual slope of 0.0047 (*p* = 0.775), and lymph node staging (pN) decreased by 0.0305 per year (*p* = 0.001). Clinical cUICC staging over the 13-year study span increased by 0.0059 per year (*p* = 0.539). During this time span, pathological (pUICC) staging decreased by a slope of 0.0298 (*p* = 0.487). Regarding tumor grading, the regression line remained unchanged over the years (*p* = 0.611).

### 3.6. Does the Radiochemotherapy Delivered to the Patient Cohort Change over Time?

**Figure 6:**
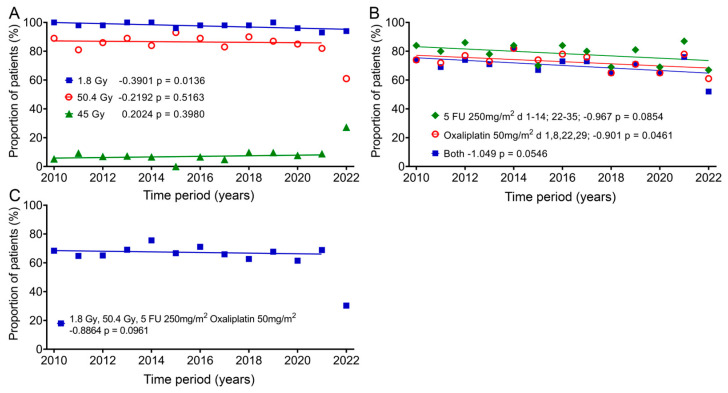
Changes in the individual groups over time for fraction dose, total dose and chemotherapy. Means and regression lines of (**A**) the percentage of patients receiving a fractionated dose of 1.8 Gy (blue squares) and a total dose of 45 Gy (green diamonds) or 50.4 Gy (red circles). (**B**) Patients receiving a 5-FU chemotherapy dose of 250 mg/m^2^ between days 1–14 and days 22–35 (green diamonds) or 50 mg/m^2^ oxaliplatin on days 1, 8, 22 and 29 (red circles), or the subgroup which received both (blue squares). (**C**) Patients receiving a fractionated therapy with 1.8 Gy up to a total dose of 50.4 Gy and 5-FU 250 mg/m^2^ and 50 mg/m^2^ oxaliplatin. The outliers in (**A**,**C**) were removed from the regression calculation. The first number after the designation indicates the slope of the regression per year, and the second number the significance of the correlation.

In addition, we also studied the change in therapy over the 13 years, as it could have a significant impact on quality of life. Almost all patients (mean: 97.6%; ± standard deviation: 2.1%) received fraction doses of 1.8 Gy, which decreased slightly by 5.1% over time according to the regression line. One group (8.5% ± 6.0%) received 45 Gy as the total dose, and there was an increase of 2.6%, and most patients (84.6% ± 7.6%) received a total dose of 50.4 Gy, with a decrease of 2.85% over the 12 years. In 2022, only 61% of patients received 50.4 Gy and 27.3% received a total dose of 45 Gy (Figure 6A). The most commonly given chemotherapy methods (78.4% ± 6.9%) were 5-FU 250mg/m^2^ administered as a continuous infusion between days 1–14 and days 22–35 and 50mg/m^2^ oxaliplatin (72.7% ± 5.8%) on days 1, 8, 22 and 29. The regression showed decreases of 12.6% and 11.7%. Both drugs were used slightly less frequently (70.1% ± 7.0%) and decreased over time by 13.6% (Figure 6B). The combination of 1.8 Gy fraction dose, 50.4 Gy total dose and 5-FU and oxaliplatin (64.4% ± 10.5%) changed only slightly over time by −2.8% (Figure 6C). Here, again, far fewer patients (30.3%) were treated with this regimen in 2022.

## 4. Discussion

When undergoing radiochemotherapy (RCT) and surgery in advanced rectal cancer (RC) therapy, symptoms and functional problems arise for patients. After collecting data for 13 years from May 2010 to September 2022, our interest was in analyzing patients’ QOL within this timeframe.

### 4.1. The Changes in QOL Scores After the RCT and 1 Year After the End of Treatment

Preoperative radiochemotherapy for advanced rectal cancer is expected to cause acute side effects that may affect the bowel and bladder, in addition to general side effects due to the systemic effects of radiation and chemotherapy, which mostly occur at the end of treatment. As expected, the greatest impact of treatment on quality of life occurred at the end of RCT and was found for diarrhoea (+24.8 pp), appetite loss (+21.4 pp), pain (+19.8 pp), role functioning (−18.9 pp) and fatigue (+16.1 pp). In contrast, there are outcomes that were influenced by RCT to a limited extent, both at the end of RCT and at the 1-year follow-up. These include dyspnoea, constipation, cognitive functioning and financial difficulties.

A very important finding was that one year after the end of therapy, all QOL scores, except body image, at least returned to levels equal to those pre-therapy, but some were even better than before: significant improvements compared to the pre-therapy values were found in the scores for weight loss (−13.4 pp) and future perspective (+15.0 pp). The only score with a distinct decrease since the beginning of therapy for RC was body image (−12.3 pp), indicating that body image issues, which affect both physical and mental health, might not receive the necessary attention. There are studies that show that cancer patients show diminished body image, without an association with the type of cancer, compared to the general population [19,20]. There are many factors that can lead to body image disturbance, like the feeling of lacking control over the body, scars and dissatisfaction with one’s shape and weight. Therefore, self-protecting or coping strategies for affected individuals, as well as encouraging self-acceptance, should be considered.

### 4.2. The Deterioration of Baseline Quality of Life with Time

Changes in individual scores over time from 2010 to 2022 were examined, in addition to an overall QOL analysis for all patients undergoing treatment for advanced rectal cancer. A completely unexpected change was the change in the slope of the baselines, and thus, worsening of the quality of life scores for role functioning, social functioning, body image, fatigue, nausea and vomiting as a function of time. Older patients [21] or patients with more advanced tumors [22] may experience a deterioration in quality of life over time. However, we were able to show that age, TNM staging, UICC staging and grading did not change. Only the regional lymph nodes showed clinical deterioration of the stage. However, there was no correlation with the five scores mentioned above. At the same time, there was an improvement in the pathological assessment of the affected lymph nodes. Both of these changes indicate a more effective therapy, but do not explain the deterioration of the five scores.

This prompted us to compare the quality of life of our cancer patients with that of the general population in Germany to see if there was a general deterioration in quality of life. There are three studies on the QLQ-C30 questionnaire for the general German population (gGp) that were conducted in 1998, 2012 and 2017. In a direct comparison of two groups, the general population and rectal cancer patients, the average difference in 2012 was 12.7 pp (standard deviation 6.4 pp), and it was 11.2 pp (SD 10.8 pp) in 2017. Rectal cancer patients are therefore clearly worse off in terms of function and symptoms than the general German population (Table 2).

The methodological circumstance of the baseline values in cancer patients used in our study being gathered post-diagnosis, when individuals may already experience symptoms, as well as being psychologically impacted by the cancer diagnosis, is crucial to remember. In addition, when the QOL data of the “healthy” reference population are compared to the QOL data of well-defined groups of patients, it must be kept in mind that this sample of the population shows a morbidity pattern that should reflect the frequency and the morbidity pattern in the current German population [23].

We then looked at changes between the 2012 and 2017 surveys. Nolte et al. [24] mention a striking deterioration in scores for the German general population analyzed in 2017 compared to data from 1998 by Schwarz and Hinz [21] and in 2012 by Hinz et al. [25]. These findings of lower functional and higher symptom scores, by an average of 9.7 pp (SD 3.2), for the EORTC QLQ-Q30 are observed for all 15 scores. Given this development in the general population, the shifts in baseline over time that occurred in our study might not exclusively be a phenomenon in rectal cancer patients. Scores that stood out with differences of >10 pp between 2012 and 2017 when comparing the general German population to the rectal cancer group were role functioning and social functioning. Other than that, the differences for rectal cancer patients between 2012 and 2017 were smaller in nine scores than for the general population, with an average deterioration of 8.2 pp (SD 8.0).

**Table 2:** EORTC QLQ-C30 mean scores of studies representing general German population compared to mean scores of rectal carcinoma patients in this study.

	General German Population				Rectal Cancer Patients			RC-gGp		1-Year Follow-Up	
	1998 [21]	2012 [25]	2017 [24]	Change 2012–2017	2012 (This Study)	2017 (This Study)	Change 2012–2017	2012	2017	Mean RC (This Study)	Mean RC-2017 gGp
**Functional scores**											
Physical	90.5	92.7	82.8	−9.9	85.4	73.9	−11.5	−7.3	−8.9	77.6	−5.2
Role	88.5	90.8	80.8	−10	75.7	52.1	−23.6	−15.1	−28.7	61.0	−19.8
Emotional	79.2	83.7	73.9	−9.8	68.7	62.2	−6.5	−15.0	−11.7	69.5	−4.4
Cognitive	91.5	93.8	83.9	−9.9	89.2	83.3	−5.9	−4.6	−0.6	79.6	−4.3
Social	91.5	93.6	84.8	−8.8	69.8	46.5	−23.3	−23.8	−38.3	60.2	−24.6
Global health status	71.5	75.9	67.0	−8.9	64.6	47.2	−17.4	−11.3	−19.8	62.4	−4.6
**Symptom scores**											
Fatigue	16.6	15.0	31.5	−16.5	29.4	40.7	−11.3	−14.4	−9.2	32.4	−0.9
Nausea/vomiting	2.7	2.1	6.0	−3.9	2.3	9.7	−7.4	−0.2	−3.7	6.2	−0.2
Pain	14.6	15.8	27.6	−11.8	23.4	22.9	+0.5	−7.6	+4.7	23.9	+3.7
Dyspnoea	7.8	6.9	18.7	−11.8	24.3	26.4	−2.1	−17.4	−7.7	22.7	−4.0
Insomnia	15.5	11.7	27.6	−15.9	35.1	31.9	+3.2	−23.4	−4.3	27.7	−0.1
Appetite loss	5.2	3.6	10.1	−6.5	10.8	15.3	−4.5	−7.2	−5.2	10.8	−0.7
Constipation	3.4	2.1	9.6	−7.5	15.3	12.5	+2.8	−13.2	−2.9	12.5	−2.9
Diarrhoea	2.9	2.5	10.4	−7.9	20.7	27.8	−7.1	−18.2	−17.4	32.0	−21.6
Financial difficulties	5.6	4.7	11.3	−6.6	15.8	25.0	−9.2	−11.1	−13.7	27.6	−16.3

General German population = normative data from the three German studies of the QLQ-C30 questionnaire; RC = rectal cancer patients = data from the study conducted here; Change 2012–2017 = the difference between 2017 and 2012 data for functional scores and the 2012 and 2017 data for symptom scores (negative values indicate a decrease in QOL from 2012 to 2017); RC-gGp = the difference between the scores of the general German population and this study for years 2012 and 2017 (negative values indicate deteriorated values in this study); Mean RC-2017 gGp = the difference between data from the general German population and the study conducted here. The data from this study were subtracted from the gGp (general German population) data for the functional scores and vice versa for the symptom scores. Negative values indicate a decrease in QOL in this study.

### 4.3. Is There an Improvement in Quality of Life over Time?

One question for this study was whether the improvements in rectal cancer treatment over the 13 years would improve patients’ quality of life. Overall, there were minor improvements in the 1-year follow-up, but also no changes or even deteriorations, and therefore, no improvement can be assumed. Overall, an improvement in therapy in terms of a reduction in side effects and, thus, an improvement in quality of life could not be proven. In addition, when comparing the one-year follow-up with the general German population, there are only minor differences in most scores, even though these were cancer patients with very advanced disease (Table 2). The only relevant impaired functional scores compared to the gGp are role functioning (19.8 pp) and social functioning (24.6 pp). Diarrhoea (21.6 pp) and financial difficulties (16.3 pp) are the symptom scores that are significantly impaired. Diarrhoea is one of the specific consequences of radiation therapy. Since little has changed in the treatment of rectal cancer over the 13 years regarding radiotherapy and chemotherapy (Figure 6), this is perhaps to be expected. The small differences that exist compared to the general German population are another reason why quality of life cannot improve much, since quality of life is already quite good for patients with advanced cancer. These surprising findings are based on just this study, and it would therefore be important to repeat these quality-of-life studies at other hospitals.

The limitations of this study are that, although a large number of patients were surveyed, only about 50 patients were surveyed per year, and not every patient took part in every survey in this study.

Another clear limitation is that the results can only partially be compared to the general population of Germany, due to a lack of normative data from the past five years. Therefore, it can be questioned whether the Nolte and Waldmann data still reflect the situation in Germany today. In general, it is quite difficult to compare our data with more recent data from other countries because of the lack of a common sampling methodology across studies. Therefore, a study conducted by Nolte et al. in 2019 examined normative data from the general population using a common sampling strategy across 15 countries, which ensured uniformity in data collection. One of the findings of this study was that inter-country comparisons between the lowest- and highest-scoring nations exceeded 10 points, indicating notable variations in global health/QOL scores among different countries [26]. Another reason for the limited comparability, specific to German normative data, may be problems in translating the questionnaires. A German study systematically questioned existing response options for the German version of the QLQ-C30 4-point response scale. The findings after a head-to-head comparison between two questionnaire versions were that by changing the German response option for “3 = quite a bit”, thus giving it a clearer meaning and creating a more adequate option, it made “4 = very much” less attractive for patients [27]. One could speculate that a cancer diagnosis and radiochemotherapy make the scores particularly sensitive to biased wording. The same study showed that the highest proportions of significant differences, due to the change in the response option, were found for the scores for physical functioning, followed by appetite loss, role functioning, emotional functioning and fatigue. This supports some of the results of our study, where all patients participating from 2010 to 2022 scored worse after RCT compared to baseline in appetite loss and fatigue for symptom scores and physical functioning and role functioning for functional scores. However, the score for emotional functioning in our study did not stand out as much in this case.

### 4.4. Factors Influencing Quality of Life

The term “health-related quality of life” (HRQOL) was introduced to specifically address the effects of health, illness and treatment on quality of life. Therefore, it excludes aspects of QOL that are not connected to health, such as cultural, political or societal attributes or the quality of the environment, public safety, education, standard of living, political freedom or cultural amenities. Unfortunately, it must be recognized that it can be challenging to distinctly separate health-related and non-health-related quality of life [7]. In addition, the construct of HRQOL can be described as very multidimensional, covering symptoms, the side effects of treatment, general health perceptions and life satisfaction, assessed through patient-reported outcomes. For a broader understanding of HRQOL beyond biological factors and symptoms, the conceptual model by Wilson and Cleary offers valuable perspectives. Their model concentrates on relationships between the different health domains, drawing a linear sequence of causal relationships along a causal pathway, starting with a biological and physiological level and moving outwards to a subjective level and individuals’ social interactions [28,29]. Linking biological and psychosocial factors might aid in understanding all aspects of well-being, which are influenced by ongoing changes in healthcare, health status and social support [28,30].

In this manner, a very prominent event was the COVID-19 pandemic, which severely impaired people’s quality of life in the social, physical, mental and economic areas worldwide from 2020 to 2023. Not only did COVID-19 survivors face physical and mental health challenges following infection, but individuals who did not contract the virus were also still vulnerable to mental health issues due to social isolation, uncertainty over health status and pandemic-related fears [31]. During that time, rectal cancer patients experienced a decline in quality of life, with functional scores like role functioning and body image being negatively impacted. Symptoms such as insomnia, defecation problems and weight loss were perceived more prominently [32]. These effects could have been caused by the threat of infection, the restrictions imposed by policymakers or a combination of these factors.

Another increasingly recognized public health concern that can lead to substantial functional impairment and reduced quality of life for affected individuals is mental disorders, among which depression and anxiety are most common. Depression does affect several dimensions of HRQOL, including emotional, physical and social well-being. Since depression cannot easily be clinically attributed to physiological abnormalities, clinicians should be attentive to signs of depression, as it is a major issue in health-related QOL [28]. Individuals diagnosed with cancer experience persistent poor health and well-being following cancer and cancer treatment [33,34]. Stressful life events, such as a cancer diagnosis, can be a pathogenic factor of mental disorders [35]. As stated previously, the data used for the analysis of the baselines reflect patients’ QOL post-diagnosis. Furthermore, the questionnaires were not designed for an investigation of signs and symptoms of mental health disorders. However, a few overlaps in symptoms can be addressed: depression can show symptoms like fatigue, changes in appetite or weight and sleep disturbances such as insomnia or hypersomnia, physical pains, digestive problems, withdrawal from social activities, and problems with sexual desire and performance. A significant increase in baseline from 2010 to 2022 presents considerably worse symptom scores for fatigue, role functioning and social functioning. The data for sexual functioning are contradictory, as our study showed significant improvement in the baseline of sexual functioning. Moderate changes (between 5 and 10 pp) in the baseline unveil worse scores in physical functioning, pain, dyspnoea, appetite loss and future perspective. Weight loss in our study tended to increase, although general weight changes cannot be considered, as weight gain was not surveyed. The same applies to sleep disturbances, as the symptom insomnia experienced minor changes in its baseline; however, hypersomnia was not considered in the questionnaires.

Although patient-reported outcomes are becoming increasingly important in medicine, it must be acknowledged that patients’ self-reporting could include potential bias. Therefore, expectations about their treatment outcomes might have influenced the patients’ answers on the questionnaires and underline the subjectiveness of QOL.

When bearing in mind the influences mentioned above affecting the mean baseline scores of rectal cancer patients, the effects during therapy need to be further evaluated. This was achieved by interpreting the results of questionnaire 3 at the end of RCT on day 35 of treatment: 20 out of 24 scores showed an approximation of the regression lines for baseline and the end of RCT. The only scores in which these regression lines diverged were the scores for future perspective, dyspnoea, insomnia and diarrhoea. A strikingly significant approximation of the regression lines for baseline and the end of RCT was seen for the scores for role functioning, social functioning, fatigue, body image and chemotherapy side effects, indicating generally worse QOL over time, while simultaneously stating a relatively smaller impact of radiochemotherapy on patients’ QOL from 2010 to 2022. Financial difficulties is the only score that significantly improved by the end of RCT; however, at the same time, it scored significantly worse at the 1-year follow-up from 2010 to 2022. These two extremes might be explained by the fact that financial difficulties become less prominent when patients are more attentive to their health [36]. At the 1-year follow-up, these financial difficulties are prominent again and, according to our study, significantly more prominent over time, while the baseline remains relatively steady.

## 5. Conclusions

An improvement in therapy in terms of a reduction in side effects and, thus, an improvement in quality of life over time could not be proven. However, the differences between the 1-year follow-up data and the normative data are small. The deterioration in individual scores over time does not appear to be a problem specific to rectal cancer patients, but rather, associated with social developments or systemic healthcare factors in German society that are not directly related to oncological diseases.

## Figures and Tables

**Table 1:** Descriptive statistics of clinical characteristics of surveyed patients from 2010 to 2022.

		2010	2011	2012	2013	2014	2015	2016	2017	2018	2019	2020	2021	2022	Total
Patients N (%)		20 (4)	54 (10)	43 (8)	55 (11)	45 (9)	27 (5)	45 (9)	41 (8)	51 (10)	31(6)	25 (5)	46 (9)	33 (6)	516
Mean age (years)		62.7	62.1	63.8	64.0	65.1	63.2	64.3	66.5	60.2	62.0	61.1	65.0	66.0	63.6
Age range (years)		46–76	23–85	32–81	23–85	38–86	38–86	31–87	50–90	36–80	15–82	41–79	44–88	40–93	15–93
Male N (%)		12 (60)	35 (65)	31 (72)	41 (75)	34 (76)	21 (78)	37 (82)	31 (76)	32 (63)	21 (68)	17 (68)	29 (63)	16 (48)	357 (69)
Female N (%)		8 (40)	19 (35)	12 (28)	14 (25)	11 (24)	6 (22)	8 (18)	10 (24)	19 (37)	10 (32)	8 (32)	17 (37)	17 (52)	159 (31)
TNM Staging N (%)															
cT	1	0 (0)	0 (0)	0 (0)	1 (2)	0 (0)	0 (0)	1 (2)	0 (0)	3 (6)	0 (0)	0 (0)	2 (4)	1 (3)	8 (2)
	2	6 (30)	5 (9)	6 (14)	4 (7)	1 (2)	3 (11)	4 (9)	5 (12)	2 (4)	1 (3)	5 (20)	5 (11)	3 (9)	50 (10)
	3	9 (45)	35 (65)	26 (60)	35 (64)	28 (62)	20 (74)	27 (60)	26 (63)	40 (78)	19 (61)	15 (60)	26 (57)	14 (42)	320 (62)
	4	5 (25)	14 (26)	11 (26)	15 (27)	16 (36)	4 (15)	13 (29)	10 (24)	6 (12)	11 (35)	5 (20)	13 (28)	15 (45)	138 (27)
pT	0	4 (20)	3 (6)	1 (2)	6 (11)	5 (11)	3 (11)	13 (29)	3 (7)	4 (8)	5 (16)	6 (24)	0 (0)	3 (9)	56 (11)
	1	1 (5)	2 (4)	3 (7)	5 (9)	2 (4)	2 (7)	2 (4)	4 (10)	2 (4)	4 (13)	2 (8)	4 (9)	0 (0)	33 (6)
	2	6 (30)	13 (24)	8 (19)	15 (27)	11 (24)	7 (26)	6 (13)	10 (24)	13 (25)	4 (13)	7 (28)	9 (20)	5 (15)	114 (22)
	3	3 (15)	24 (44)	20 (47)	16 (29)	14 (31)	13 (48)	15 (33)	12 (29)	21 (41)	13 (42)	7 (28)	22 (48)	10 (30)	190 (37)
	4	3 (15)	4 (7)	6 (14)	2 (4)	5 (11)	0 (0)	2 (4)	5 (12)	2 (4)	2 (6)	0 (0)	4 (9)	2 (6)	37 (7)
Unknown		3 (15)	8 (15)	5 (12)	11 (20)	8 (18)	2 (7)	7 (16)	7 (17)	9 (18)	3 (10)	3 (12)	7 (15)	13 (39)	86 (17)
cN	0	6 (30)	12 (22)	12 (28)	13 (24)	7 (16)	1 (4)	8 (18)	14 (34)	8 (16)	5 (16)	4 (16)	11 (24)	6 (18)	107 (21)
	1	5 (25)	21 (39)	16 (37)	26 (47)	23 (51)	15 (56)	13 (29)	12 (29)	25 (49)	12 (39)	10 (40)	16 (35)	11 (33)	205 (40)
	2	7 (35)	7 (13)	4 (9)	12 (22)	9 (20)	6 (22)	16 (36)	11 (27)	13 (25)	11 (35)	7 (28)	16 (35)	15 (45)	134 (26)
	3	0 (0)	0 (0)	0 (0)	0 (0)	0 (0)	0 (0)	0 (0)	0 (0)	0 (0)	0 (0)	0 (0)	0 (0)	0 (0)	0 (0)
Unknown		2 (10)	14 (26)	11 (26)	4 (7)	6 (13)	5 (19)	8 (18)	4 (10)	5 (10)	3 (10)	4 (16)	3 (7)	1 (3)	70 (14)
pN	0	15 (75)	26 (48)	22 (51)	28 (51)	24 (53)	14 (52)	27 (60)	26 (63)	35 (69)	21 (68)	18 (72)	26 (57)	13 (39)	295 (57)
	1	2 (10)	8 (15)	9 (21)	12 (22)	9 (20)	9 (33)	8 (18)	6 (15)	6 (12)	6 (19)	4 (16)	8 (17)	7 (21)	94 (18)
	2	0 (0)	12 (22)	7 (16)	4 (7)	4 (9)	2 (7)	3 (7)	2 (5)	1 (2)	1 (3)	0 (0)	3 (7)	0 (0)	39 (8)
	3	0 (0)	0 (0)	0 (0)	0 (0)	0 (0)	0 (0)	0 (0)	0 (0)	0 (0)	0 (0)	0 (0)	0 (0)	0 (0)	0 (0)
Unknown		3 (15)	8 (15)	5 (12)	11 (20)	8 (18)	2 (7)	7 (16)	7 (17)	9 (18)	3 (10)	3 (12)	9 (20)	13 (39)	88 (17)
cM	0	15 (75)	33 (61)	26 (60)	37 (67)	30 (67)	19 (70)	35 (78)	31 (76)	42 (82)	23 (74)	18 (72)	35 (76)	25 (76)	369 (72)
	1	3 (15)	7 (13)	7 (16)	13 (24)	11 (24)	4 (15)	3 (7)	5 (12)	5 (10)	5 (16)	6 (24)	10 (22)	7 (21)	86 (17)
Unknown		2 (10)	14 (26)	10 (23)	5 (9)	4 (9)	4 (15)	7 (16)	5 (12)	4 (8)	3 (10)	1 (4)	1 (2)	1 (3)	61 (12)
UICC Staging N (%)															
c	I	1 (5)	2 (4)	2 (5)	3 (5)	0 (0)	0 (0)	3 (7)	1 (2)	3 (6)	0 (0)	2 (8)	2 (4)	2 (6)	21 (4)
	II	3 (15)	10 (19)	8 (19)	8 (15)	5 (11)	1 (4)	5 (11)	10 (24)	4 (8)	5 (16)	1 (4)	7 (15)	3 (9)	70 (14)
	III	8 (40)	20 (37)	16 (37)	25 (45)	23 (51)	18 (67)	24 (53)	19 (46)	34 (67)	17 (55)	15 (60)	24 (52)	19 (58)	262 (51)
	IV	3 (15)	8 (15)	6 (14)	13 (24)	11 (24)	4 (15)	3 (7)	5 (12)	5 (10)	5 (16)	5 (20)	10 (22)	6 (18)	84 (16)
Unknown		5 (25)	14 (26)	11 (26)	6 (11)	6 (13)	4 (15)	10 (22)	6 (15)	5 (10)	4 (13)	2 (8)	3 (7)	3 (9)	79 (15)
p	I	5 (25)	10 (19)	8 (19)	13 (24)	4 (9)	4 (15)	8 (18)	12 (29)	13 (25)	6 (19)	8 (32)	8 (17)	2 (6)	101 (20)
	II	4 (20)	13 (24)	12 (28)	8 (15)	10 (22)	7 (26)	7 (16)	8 (20)	15 (29)	10 (32)	4 (16)	12 (26)	4 (12)	114 (22)
	III	2 (10)	12 (22)	10 (23)	10 (18)	10 (22)	9 (33)	7 (16)	7 (17)	7 (14)	3 (10)	3 (12)	6 (13)	2 (6)	88 (17)
	IV	3 (15)	7 (13)	7 (16)	5 (9)	5 (11)	1 (4)	2 (4)	4 (10)	3 (6)	4 (13)	1 (4)	10 (22)	4 (12)	56 (11)
Unknown		6 (30)	12 (22)	6 (14)	19 (35)	16 (36)	6 (22)	21 (47)	10 (24)	13 (25)	8 (26)	9 (36)	10 (22)	21 (64)	157 (30)
Grading N (%)															
	1	2 (10)	1 (2)	1 (2)	1 (2)	2 (4)	1 (4)	0 (0)	4 (10)	3 (6)	1 (3)	0 (0)	2 (4)	2 (6)	20 (4)
	2	12 (60)	40 (74)	34 (79)	37 (67)	32 (71)	24 (89)	32 (71)	32 (78)	40 (78)	20 (65)	19 (76)	36 (78)	22 (67)	380 (74)
	3	3 (15)	7 (13)	7 (16)	11 (20)	9 (20)	1 (4)	7 (16)	1 (2)	7 (14)	6 (19)	4 (16)	6 (13)	5 (15)	74 (14)
	4	0 (0)	0 (0)	0 (0)	0 (0)	0 (0)	0 (0)	0 (0)	0 (0)	0 (0)	0 (0)	0 (0)	0 (0)	0 (0)	0 (0)
Unknown		3 (15)	6 (11)	1 (2)	6 (11)	2 (4)	1 (4)	6 (13)	4 (10)	1 (2)	4 (13)	2 (8)	2 (4)	4 (12)	42 (8)

The prefix c is used to describe the stage before treatment based on information from clinical examination, imaging, endoscopy and biopsy, while p describes the stage after neoadjuvant treatment and histopathologic examination of the surgical specimen.

## Data Availability

The data that support the findings of this study are available from the corresponding author upon reasonable request.
